# Challenges in agrochemicals design

**DOI:** 10.1186/1758-2946-5-S1-O17

**Published:** 2013-03-22

**Authors:** KJ Schleifer

**Affiliations:** 1 BASF SE, Ludwigshafen, Germany

## 

Design of active ingredients is a multidimensional task. Sufficient target activity in combination with high bioavailability and no (or at least low) toxicological behaviour are prerequisites for promising candidates. In this respect, pharmaceutical and agrochemical companies need to address the same issues. Considering the target activity one can generally conclude that "target is target" regardless of whether the respective organism is a human or a pest. In detail however, drugs modulate activity of dysfunctional proteins or their functional counterparts aiming to reduce the pathogenic effects. In contrast to that agrochemicals modulate vitally important proteins in order to keep harmful organisms under control.

Bioavailability of drugs usually addresses the human patient with regard to a preferred oral application. Agrochemicals have to be divided into herbicides, fungicides and insecticides controlling weed, harmful fungi and insect pests. For each of these harmful organisms different absorption, distribution, metabolism and excretion routes have to be considered leading to quite different physicochemical properties of lead compounds.

Based on the above-mentioned similarities and differences, the presentation will give an overview on commonly applied computational approaches in pharmaceutical and agrochemical companies as well as several unique challenges relevant just for the design of potent crop protection compounds.

